# Modeling and Experimental Study of Vibration Energy Harvester with Triple-Frequency-Up Voltage Output by Vibration Mode Switching

**DOI:** 10.3390/mi15081013

**Published:** 2024-08-06

**Authors:** Jiawen Xu, Zhikang Liu, Wenxing Dai, Ru Zhang, Jianjun Ge

**Affiliations:** 1 Jiangsu Key Lab of Remote Measurement and Control, School of Instrument Science and Engineering, Southeast University, Nanjing 210096, China; 2 Institute of Biomedical Devices, Southeast University, Suzhou 215163, China

**Keywords:** vibration energy harvesting, stopper, vibration mode switching, frequency up-conversion

## Abstract

Conventional wireless sensors rely on chemical batteries. Replacing or charging their batteries is tedious and costly in some situations. As usable kinetic energy exists in the environment, harvesting vibration energy and converting it into electrical energy has become a hotspot. However, the power output capability of a conventional piezoelectric energy harvester (PEH) is limited by its low operational frequency. This paper presents a new mechanism for achieving continuous triple-frequency-up voltage output in a PEH. The proposed system consists of a slender piezoelectric cantilever with two short cantilever-based stoppers. The piezoelectric cantilever undergoes a pure bending mode without contacting the stoppers. In addition, the beam switches into a new vibration mode by contacting the stoppers. The vibration modes switching yields reverses the signs of voltage outputs, inducing triple-frequency-up voltage output. Analytical and experimental investigations are presented, and it is shown that a significant triple-frequency up-conversion of the voltage output can be obtained over a wide frequency range. A peak power output of 3.03 mW was obtained. The proposed energy harvester can support a wireless sensor node.

## 1. Introduction

Wireless and remote sensor nodes have received significant attention due to the growth of the Internet of Things (IoT) [1,2,3,4,5]. IoT sensor nodes usually rely on chemical batteries. However, the replacement of batteries in some situations is tedious and expensive. In recent decades, harvesting ambient environmental energy and converting it into electricity has emerged as a promising solution for powering sensor nodes [1,4,5]. Mechanical vibration is ubiquitous and will not be affected by sunshine conditions. Among the mechanisms used to harvest vibration energy, piezoelectric energy harvesting has the advantages of a simple structure and high efficiency [6,7,8,9].

Typically, a piezoelectric vibration energy harvester (PEH), consisting of a piezoelectric cantilever with proof mass, is a second-order dynamics system [6,7,8,9]. This second-order system operates optimally when its resonant frequency equals that of the excitation. On the other hand, a slightly excited frequency dis-matching yields a drastic decrease in power output [8,9,10,11,12]. In addition, piezoelectric transducers have low power output capabilities. This is because a piezoelectric transducer has the inherent characteristic of a capacitor with a large impedance in the low-frequency range [13,14,15]. Considering that environmental vibrations exist over a wide frequency range, widening bandwidth and up-converting the power output frequency has been focused on. To expand energy harvesters’ bandwidth, manual resonant frequency adjustment [10], Duffing-type nonlinearity with cubic nonlinear terms [16,17,18,19,20,21,22,23,24,25,26,27,28,29], stopper-based segmental stiffness [11], 1:2 internal resonance [30,31,32,33,34], multimodal configuration [35,36], etc., were proposed. The Duffing-type PEHs have been investigated intensively [13,14,15,16,17,18,19,20,21,22,23,24,25]. In particular, a bistable PEH vibrating between two attractors would have significantly enlarged bandwidth [20,21,22,23,24,25]. Since then, small potential barriers, multiple stable potential barriers, and flexible potential barriers have been proposed and analyzed [26,27,28,29].

Though the broadband energy harvesting is promising, the PEHs would suffer from limited power output capability [37,38,39,40,41]. This can be attributed to the fact that piezoelectric transducers have a large impedance in the low-frequency range [13,14,15]. In such a case, increasing the power output requires extremely large amplitude voltage output due to the large matching impedance in the external circuit. On the other hand, piezoelectric transducers possess strain/electric field limitations and thus the harvester cannot output very large voltages in the low-frequency range. An effective solution is increasing the frequency of the voltage output and reducing the impedance of the piezoelectric transducer [13,14,15]. Therefore, frequency up-conversion mechanisms are proposed. The conventional frequency up-converting mechanisms absorb mechanical energy from low-frequency vibrations and transfer it into a high-frequency oscillator for frequency up-conversion. Impact was adopted for energy transmission and high-ratio frequency up-converting [42,43,44,45,46,47]. Specifically, a low-frequency component absorbs the vibration energy, and a high-frequency harvester converts the mechanical energy into electrical energy. The low- and high-frequency components are coupled by the impact motion or magnetic coupling. For instance, Renaud proposed an impact-based frequency up-conversion mechanism [42]. Gu illustrated that the energy conversion efficiency of a frequency-up harvester can be enhanced by reducing the mechanical loss [43]. In addition, a hung mass was introduced to trigger frequency up-conversion through a hitting motion [44]. On the other hand, the impact between the low- and high-frequency components occurs within a very short period. In other words, a conventional system has diminishing up-converted output or does not demonstrate voltage output most of the time; therefore, its efficiency can be further improved.

In addition, stopper-based PEHs also feature frequency up-conversion due to impact motion. Ashraf analyzed the system dynamics of a frequency up-conversion PEH with mechanical stoppers [45]. Halim adopted a flexible stopper to form a stopper-based harvester. It was demonstrated that the increased effective stiffness also broadened the bandwidth [46]. In conventional frequency up-conversion mechanisms, a flexible beam is required to absorb the low-frequency vibrations and hit the high-frequency harvester. In other words, the impact and the mechanical energy transfer occur within a very short hitting period [43], which reduces the overall efficiency.

An ideal frequency-up-based PEH should up-convert the output in a continuous manner. In this study, we proposed and analyzed a new mechanism that takes advantage of vibration mode switching. In particular, the piezoelectric cantilever undergoes a pure bending mode without contacting the stoppers. In addition, the beam switches to a new vibration mode with contacting the stoppers. Multiple-time reversing of the sign of the voltage output leads to a triple-frequency up-conversion of voltage output. The remainder of this paper is organized as follows: The concept and system configurations are presented in Section 2. A detailed derivation of the vibration modes is presented in Section 3. Case studies and discussions of the frequency up-converting performance of the system are presented in Section 4. Finally, the conclusions are provided in Section 5.

## 2. System Configuration and Operational Principle

**Figure 1:**
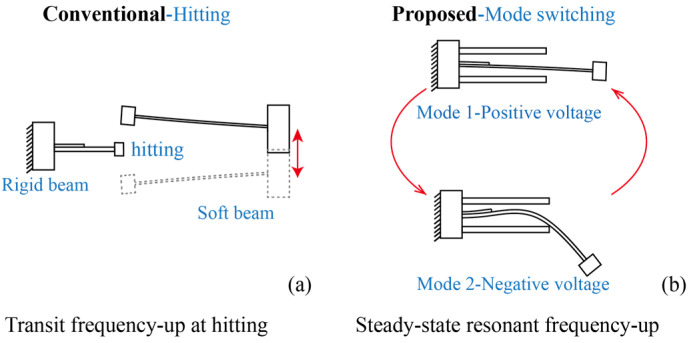
(**a**) Conventional frequency up-conversion mechanism by hitting (arrow: vibration direction); (**b**) proposed frequency up-conversion mechanism by mode switching (arrow: mode switching).

In this section, we illustrate the design concept of the proposed system, as shown in Figure 1. The conventional frequency up-converting PEH is shown in Figure 1a. It contains a soft beam that absorbs the energy, and a rigid piezoelectric beam converts the mechanical energy into electrical energy. The rigid piezoelectric beam has a relatively higher resonant frequency than that of the soft one. The soft beam absorbs low-frequency vibrational energy and triggers contact with the rigid beam, leading to two-beam contact. The triggering occurs within a very short time. The frequency up-conversion in a conventional PEH is essentially a transit motion within a very short period, i.e., the system shows diminished frequency up-converted output or does not have voltage output most of the time.

The proposed PEH with triple-frequency up-conversion is shown in Figure 1b. The PEH comprises a long slender beam and two short/rigid beams as stoppers. The slender beam was placed at the center of the two stoppers. A copper-based proof mass was attached to the free end of the slender beam for resonant frequency adjustment and vibration energy absorption. The slender beam had dimensions of 75.18 × 20 × 0.16 mm^3^ and the two stoppers with dimensions of 45.43 × 20 × 0.6 mm^3^ were made of spring steel sheets. In addition, a one-piece piezoelectric transducer, with dimensions of 15 × 20 × 0.2 mm^3^, was glued at the root of the spring steel slender beam. The two stoppers were made of thick cantilevers with much greater stiffness. Additionally, the two stoppers were glued to the same base and placed in the upper and lower vicinity of the slender beam. In addition, the main slender piezoelectric beam hit the stopper at the middle point.

**Figure 2:**
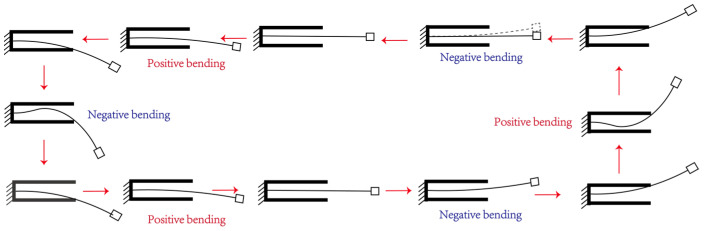
Operational principle of the frequency up-conversion.

In the proposed system, the piezoelectric cantilever has two different vibration modes. Firstly, the slender piezoelectric cantilever undergoes a pure bending mode without contacting the stoppers, as shown in Figure 1b and Figure 2. Secondly, the beam switches into a new vibration mode when contacting the stoppers, as shown in Figure 1b and Figure 2. The occurrence of the new vibration mode can be attributed to the fact that the stoppers provide a new boundary condition of constant displacement in this scenario. The additional boundary conditions yield a new bending mode of the slender piezoelectric beam. In addition, as the main slender beam is either in contact or non-contact with the stoppers, the two bending modes switch multiple times during one operation for the frequency up-conversion of voltage output.

The detailed mechanism of the triple-frequency up-conversion is shown in Figure 2. This figure shows the different bending motions of the slender beam in one circle of the resonant motion. It can be determined that the beam is in the first bending mode when the beam does not contact the stoppers. Here, the piezoelectric transducer at the root shows beading in the same direction of the moving mass. In addition, the bending shape of the slender beam switches to a complicated curved one when the main beam hits the stopper. Here, the piezoelectric transducer at the root shows beading in the opposite direction of the moving mass. Notably, the complicated bending mode shows the opposite strain distribution at the root of the beam. In this scenario, the piezoelectric transducers bonded at the root of the slender beam output the opposite voltage during the switching of the vibration mode within one resonance cycle. Thus, the piezoelectric transducer outputs three voltage cycles, yielding triple-frequency up-conversion of the voltage output. It is worth noting that the slender main beam is still working in its first resonant frequency while the voltage output is tripled. The frequency up-conversion of the voltage output stems from the reversing of local strain distribution due to different vibration modes. The frequency up-conversion of the voltage output exists along with the large amplitude resonance motion of the main beam. This phenomenon ensures the frequency up-conversion of voltage output without failure or interruptions over a long time period.

## 3. Modeling of Vibration Modes

In order to illustrate the operational principle of the piezoelectric cantilever that can facilitate vibration mode-switching-based frequency up-conversion of the voltage output, a modal analysis of the piezoelectric beam with and without contact with the stoppers is formulated. Specifically, for the given boundary conditions during the vibration, we solved the eigenvectors for the mode shapes of the slender piezoelectric beam. This is because the difference between the two vibration modes mentioned in Figure 1b stems from the boundary conditions of contact, i.e., displacement constraint, between the main beam and the two stoppers.

### 3.1. Theoretical Modeling of Vibration Mode

**Figure 3:**
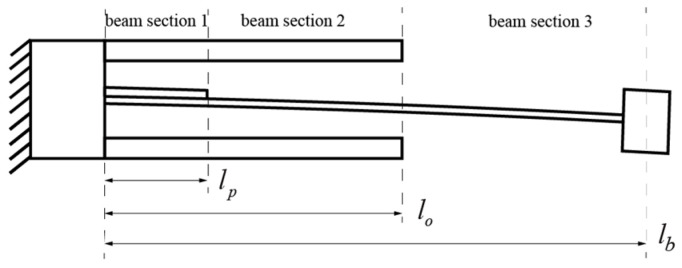
Modeling of conventional 1st bending mode.

**Figure 4:**
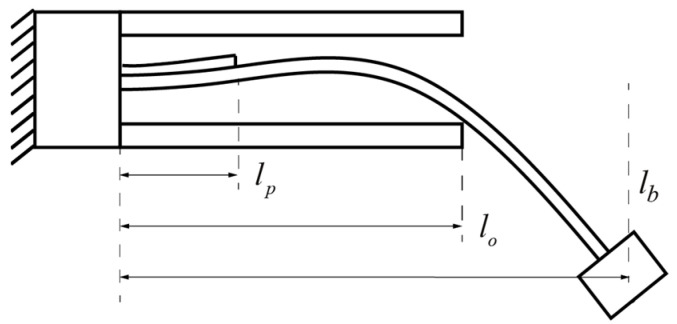
Modeling of stopper-forced vibration mode.

Euler beam theory was employed to solve the mode shape of the piezoelectric beam: (1) the effects of shear deformation and rotary inertia were neglected; (2) any section of the beam remained a flat plane after deformation; and (3) the PZT layer was perfectly bonded without shear strain between the layers. In this research, we divided the piezoelectric beam into three sections, as shown in Figure 3 and Figure 4. The first section contained a piezoelectric transducer, from *x* = 0 to *x* = *l_p_*. The second section of the beam denoted the section between the piezoelectric transducer and the hitting point, from *x* = *l_p_*$x=l_{p}$ to *x* = *l_o_*$x=l_{o}$. The last section denoted the segment of the beam between the hitting point and the free end, from *x* = *l_o_* to *x* = *l_b_*. In the following analysis, *ρA*(*x*) is the density of the beam per unit length, which remains the same in beam sections 2 and 3, the sections without the piezoelectric transducer attached, as shown in Figure 3. And in beam section 1, a piezoelectric transducer was glued. We represented the mode shapes of the beam as $\phi_{i}(x)$ (*i* = 1, 2, 3) for the beam segments in sections 1, 2, and 3, respectively [6,48],

(1a) $$\phi_{1}(x)=A_{1}\cos k_{1}x+B_{1}\sin k_{1}x+C_{1}\cosh k_{1}x+D_{1}\sinh k_{1}x$$

(1b) $$\phi_{2}(x)=A_{2}\cos k_{2}\left(x-l_{p}\right)+B_{2}\sin k_{2}\left(x-l_{p}\right)+C_{2}\cosh k_{2}\left(x-l_{p}\right)+D_{2}\sinh k_{2}\left(x-l_{p}\right)$$

(1c) $$\phi_{3}(x)=A_{3}\cos k_{2}\left(x-l_{o}\right)+B_{3}\sin k_{2}\left(x-l_{o}\right)+C_{3}\cosh k_{2}\left(x-l_{o}\right)+D_{3}\sinh k_{2}\left(x-l_{o}\right)$$

where *A_i_*, *B_i_*, *C_i_*, and *D_i_* are the coefficients of the mode shape functions. The eigenvalues are $k_{1}=\sqrt[{4}]{\omega^{2}\frac{\rho_{b}A_{b}+\rho_{p}A_{p}}{EI_{eq}}}$ and $k_{2}=\sqrt[{4}]{\omega^{2}\frac{\rho_{b}A_{b}}{EI_{b}}}$, and *EI_eq_* and *EI_b_* are the bending stiffnesses of the segments containing a piezoelectric transducer and the one with a substrate only, respectively. In addition, sections 2 and 3 have the same eigenvalue $k_{2}$ as they have the same parameters per unit length.

Firstly, we focus on the vibration mode of the 1st bending mode. In this case, the main beam does not hit the stoppers, as shown in Figure 3. The boundary conditions of the beam at the fixed end, with zero displacement and rotation, are given as follows:

(2a) $$\phi_{1}(0)=0,$$

(2b) $${\frac{d\phi_{1}(x)}{dx}|}_{x=0}=0$$

which yield

(3a) $$\phi_{1}(x)=A_{1}\cos0+B_{1}\sin0+C_{1}\cosh0+D_{1}\sinh0$$

(3b) $$\frac{d\phi_{1}(x)}{dx}=-A_{1}k_{1}\sin0+B_{1}k_{1}\cos0+C_{1}k_{1}\sinh0+D_{1}k_{1}\cosh0$$

In addition, we have the following continuity relations at the joints between sections 1 and 2, and 2 and 3, respectively:

(4a) $$\phi_{1}(l_{p})=\phi_{2}(l_{p}),$$

(4b) $${\frac{d\phi_{1}(x)}{dx}|}_{x=l_{p}}={\frac{d\phi_{2}(x)}{dx}|}_{x=l_{p}}$$

(4c) $${EI_{eq}\frac{d^{2}\phi_{1}(x)}{dx^{2}}|}_{x=l_{p}}=EI_{b}{\frac{d^{2}\phi_{2}(x)}{dx^{2}}|}_{x=l_{p}},$$

(4d) $$EI_{eq}{\frac{d^{3}\phi_{1}(x)}{dx^{3}}|}_{x=l_{p}}=EI_{b}{\frac{d^{3}\phi_{2}(x)}{dx^{3}}|}_{x=l_{p}}$$

(4e) $$\phi_{2}(l_{o})=\phi_{3}(l_{o}),$$

(4f) $${\frac{d\phi_{2}(x)}{dx}|}_{x=l_{o}}={\frac{d\phi_{3}(x)}{dx}|}_{x=l_{o}}$$

(4g) $${EI_{b}\frac{d^{2}\phi_{2}(x)}{dx^{2}}|}_{x=l_{o}}=EI_{b}{\frac{d^{2}\phi_{3}(x)}{dx^{2}}|}_{x=l_{o}},$$

which yield

(5a) $$\begin{array}{l} A_{1}\cos k_{1}l_{p}+B_{1}\sin k_{1}l_{p}+C_{1}\cosh k_{1}l_{p}+D_{1}\sinh k_{1}l_{p}\\ =A_{2}\cos0+B_{2}\sin0+C_{2}\cosh0+D_{2}\sinh0 \end{array}$$

(5b) $$\begin{array}{l} -A_{1}k_{1}\sin k_{1}l_{p}+B_{1}k_{1}\cos k_{1}l_{p}+C_{1}k_{1}\sinh k_{1}l_{p}+D_{1}k_{1}\cosh k_{1}l_{p}\\ =-A_{2}k_{2}\sin0+B_{2}k_{2}\cos0+C_{2}k_{2}\sinh0+D_{2}k_{2}\cosh0 \end{array}$$

(5c) $$\begin{array}{l} EI_{eq}\left(-A_{1}{k_{1}}^{2}\cos k_{1}l_{p}-B_{1}{k_{1}}^{2}\sin k_{1}l_{p}+C_{1}{k_{1}}^{2}\cosh k_{1}l_{p}+D_{1}{k_{1}}^{2}\sinh k_{1}l_{p}\right)\\ =EI_{b}\left(-A_{2}{k_{1}}^{2}\cos0-B_{2}{k_{2}}^{2}\sin0+C_{2}{k_{2}}^{2}\cosh0+D_{2}{k_{2}}^{2}\sinh0\right) \end{array}$$

(5d) $$\begin{array}{l} EI_{eq}\left(A_{1}{k_{1}}^{3}\sin k_{1}l_{p}-B_{1}{k_{1}}^{3}\cos k_{1}l_{p}+C_{1}{k_{1}}^{3}\sinh k_{1}l_{p}+D_{1}{k_{1}}^{3}\cosh k_{1}l_{p}\right)\\ =EI_{b}\left(A_{2}{k_{2}}^{3}\sin0-B_{2}{k_{2}}^{3}\cos0+C_{2}{k_{2}}^{3}\sinh0+D_{2}{k_{2}}^{3}\cosh0\right) \end{array}$$

(5e) $$\begin{array}{l} A_{2}\cos k_{2}\left(l_{o}-l_{p}\right)+B_{2}\sin k_{2}\left(l_{o}-l_{p}\right)+C_{2}\cosh k_{2}\left(l_{o}-l_{p}\right)+D_{2}\sinh k_{2}\left(l_{o}-l_{p}\right)\\ =A_{3}\cos0+B_{3}\sin0+C_{3}\cosh0+D_{3}\sinh0 \end{array}$$

(5f) $$\begin{array}{l} -A_{2}k_{2}\sin k_{2}\left(l_{o}-l_{p}\right)+B_{2}k_{2}\cos k_{2}\left(l_{o}-l_{p}\right)+C_{2}k_{2}\sinh k_{2}\left(l_{o}-l_{p}\right)+D_{2}k_{2}\cosh k_{2}\left(l_{o}-l_{p}\right)\\ =-A_{3}k_{2}\sin0+B_{3}k_{2}\cos0+C_{3}k_{2}\sinh0+D_{3}k_{2}\cosh0 \end{array}$$

(5g) $$\begin{array}{l} -A_{2}{k_{2}}^{2}\cos k_{2}\left(l_{o}-l_{p}\right)-B_{2}{k_{2}}^{2}\sin k_{2}\left(l_{o}-l_{p}\right)+C_{2}{k_{2}}^{2}\cosh k_{2}\left(l_{o}-l_{p}\right)+D_{2}{k_{2}}^{2}\sinh k_{2}\left(l_{o}-l_{p}\right)\\ =-A_{3}{k_{2}}^{2}\cos0-B_{3}{k_{2}}^{2}\sin0+C_{3}{k_{2}}^{2}\cosh0+D_{3}{k_{2}}^{2}\sinh0 \end{array}$$

(5h) $$\begin{array}{l} A_{2}{k_{2}}^{3}\sin k_{2}\left(l_{o}-l_{p}\right)-B_{2}{k_{2}}^{3}\cos k_{2}\left(l_{o}-l_{p}\right)+C_{2}{k_{2}}^{3}\sinh k_{2}\left(l_{o}-l_{p}\right)+D_{2}{k_{2}}^{3}\cosh k_{2}\left(l_{o}-l_{p}\right)\\ =A_{3}{k_{2}}^{3}\sin0-B_{3}{k_{2}}^{3}\cos0+C_{3}{k_{2}}^{3}\sinh0+D_{3}{k_{2}}^{3}\cosh0 \end{array}$$

The boundary conditions in Equation (4) represent continuous displacement, rotation angles, and bending moments at the joints between sections 1 and 2, and 2 and 3. Additionally, the boundary conditions at the free end with a proof mass attached are given as follows:

(6a) $$EI_{b}{\frac{d^{2}\phi_{3}(x)}{dx^{2}}|}_{x=l_{b}}=0,$$

(6b) $$EI_{b}{\frac{d^{3}\phi_{3}(x)}{dx^{3}}q(t)|}_{x=l_{b}}=M_{p}{\frac{d^{2}q(t)}{dt^{2}}\phi_{3}(x)|}_{x=l_{b}}$$

which yield

(7a) $$-A_{3}{k_{2}}^{2}\cos k_{2}\left(l_{b}-l_{o}\right)-B_{3}{k_{2}}^{2}\sin k_{2}\left(l_{b}-l_{o}\right)+C_{3}{k_{2}}^{2}\cosh k_{2}\left(l_{b}-l_{o}\right)+D_{3}{k_{2}}^{2}\sinh k_{2}\left(l_{b}-l_{o}\right)=0$$

(7b) $$\begin{array}{l} EI_{b}\left(A_{3}{k_{2}}^{3}\sin k_{2}\left(l_{b}-l_{o}\right)-B_{3}{k_{2}}^{3}\cos k_{2}\left(l_{b}-l_{o}\right)+C_{3}{k_{2}}^{3}\sinh k_{2}\left(l_{b}-l_{o}\right)+D_{3}{k_{2}}^{3}\cosh k_{2}\left(l_{b}-l_{o}\right)\right)\\ =-\omega^{2}M_{p}\left(A_{3}{k_{2}}^{3}\sin k_{2}\left(l_{b}-l_{o}\right)-B_{3}{k_{2}}^{3}\cos k_{2}\left(l_{b}-l_{o}\right)+C_{3}{k_{2}}^{3}\sinh k_{2}\left(l_{b}-l_{o}\right)+D_{3}{k_{2}}^{3}\cosh k_{2}\left(l_{b}-l_{o}\right)\right) \end{array}$$

The boundary conditions in Equation (4) indicate zero bending moments at the free end and acceleration force condition. By grouping Equations (3), (5), and (7) together, we obtain the following eigenvalue problem regarding the coefficients of the three segments of the piezoelectric beam as follows:

(8) $$d={\left[\begin{array}{cccccccccccc} A_{1} & B_{1} & C_{1} & D_{1} & A_{2} & B_{2} & C_{2} & D_{2} & A_{3} & B_{3} & C_{3} & D_{3} \end{array}\right]}^{T}$$

we have

(9) $$K_{1}\left(k_{1},k_{2}\right)d=0$$

where **K_1_** =

(10) $$\left[\begin{array}{cccccccccccc} 1 & 0 & 1 & 0 & 0 & 0 & 0 & 0 & 0 & 0 & 0 & 0\\ 0 & k_{1} & 0 & k_{1} & 0 & 0 & 0 & 0 & 0 & 0 & 0 & 0\\ \cos k_{1}l_{p} & \sin k_{1}l_{p} & \cosh k_{1}l_{p} & \sinh k_{1}l_{p} & -1 & 0 & -1 & 0 & 0 & 0 & 0 & 0\\ -k_{1}\sin k_{1}l_{p} & k_{1}\cos k_{1}l_{p} & k_{1}\sinh k_{1}l_{p} & k_{1}\cosh k_{1}l_{p} & 0 & -k_{2} & 0 & -k_{2} & 0 & 0 & 0 & 0\\ -EI_{eq}{k_{1}}^{2}\cos k_{1}l_{p} & -EI_{eq}{k_{1}}^{2}\sin k_{1}l_{p} & EI_{eq}{k_{1}}^{2}\cosh k_{1}l_{p} & EI_{eq}{k_{1}}^{2}\sinh k_{1}l_{p} & EI_{b}{k_{2}}^{2} & 0 & -EI_{b}{k_{2}}^{2} & 0 & 0 & 0 & 0 & 0\\ EI_{eq}{k_{1}}^{3}\sin k_{1}l_{p} & -EI_{eq}{k_{1}}^{3}\cos k_{1}l_{p} & EI_{eq}{k_{1}}^{3}\sinh k_{1}l_{p} & EI_{eq}{k_{1}}^{3}\cosh k_{1}l_{p} & 0 & EI_{b}{k_{2}}^{3} & 0 & -EI_{b}{k_{2}}^{3} & 0 & 0 & 0 & 0\\ 0 & 0 & 0 & 0 & \cos k_{2}\left(l_{o}-l_{p}\right) & \sin k_{2}\left(l_{o}-l_{p}\right) & \cosh k_{2}\left(l_{o}-l_{p}\right) & \sinh k_{2}\left(l_{o}-l_{p}\right) & 0 & 0 & 0 & 0\\ 0 & 0 & 0 & 0 & 0 & 0 & 0 & 0 & 1 & 0 & 1 & 0\\ 0 & 0 & 0 & 0 & -k_{2}\sin k_{2}\left(l_{o}-l_{p}\right) & k_{2}\cos k_{2}\left(l_{o}-l_{p}\right) & k_{2}\sinh k_{2}\left(l_{o}-l_{p}\right) & k_{2}\cosh k_{2}\left(l_{o}-l_{p}\right) & 0 & -k_{2} & 0 & -k_{2}\\ 0 & 0 & 0 & 0 & -{k_{2}}^{2}\cos k_{2}\left(l_{o}-l_{p}\right) & -{k_{2}}^{2}\sin k_{2}\left(l_{o}-l_{p}\right) & {k_{2}}^{2}\cosh k_{2}\left(l_{o}-l_{p}\right) & {k_{2}}^{2}\sinh k_{2}\left(l_{o}-l_{p}\right) & {k_{2}}^{2} & 0 & -{k_{2}}^{2} & 0\\ 0 & 0 & 0 & 0 & 0 & 0 & 0 & 0 & -{k_{2}}^{2}\cos k_{2}\left(l_{b}-l_{o}\right) & -{k_{2}}^{2}\sin k_{2}\left(l_{b}-l_{o}\right) & {k_{2}}^{2}\cosh k_{2}\left(l_{b}-l_{o}\right) & {k_{2}}^{2}\sinh k_{2}\left(l_{b}-l_{o}\right)\\ 0 & 0 & 0 & 0 & 0 & 0 & 0 & 0 & EI_{b}{k_{2}}^{3}\sin k_{2}\left(l_{b}-l_{o}\right)+\omega^{2}M_{p}\cos k_{2}\left(l_{b}-l_{o}\right) & -EI_{b}{k_{2}}^{3}\cos k_{2}\left(l_{b}-l_{o}\right)+\omega^{2}M_{p}\sin k_{2}\left(l_{b}-l_{o}\right) & EI_{b}{k_{2}}^{3}\sinh k_{2}\left(l_{b}-l_{o}\right)+\omega^{2}M_{p}\cosh k_{2}\left(l_{b}-l_{o}\right) & EI_{b}{k_{2}}^{3}\cosh k_{2}\left(l_{b}-l_{o}\right)+\omega^{2}M_{p}\sinh k_{2}\left(l_{b}-l_{o}\right) \end{array}\right]$$

We can obtain the characteristic equation of the piezoelectric beam by letting the determinant of **K_1_** be 0, and then solving the eigenvalues $k_{1}$ and $k_{2}$. The characteristic equation of this integrated system is a transcendental equation that yields infinitely many solutions which correspond to different eigenvalues. The solutions can be solved numerically. Furthermore, the vibration mode shapes of the beam with and without hitting the stoppers can be obtained by solving the eigenvector **d**.

In addition, we proceed to the vibration mode in the case when the main beam hits the stoppers, as shown in Figure 4. The key difference between the conventional 1st bending mode and the stopper-forced bending mode stems from the displacement limitation at the hitting point, i.e., *x* = *l*_o_$x=l_{o}$. And the distance between the stopper and the main beam is denoted as *D*_0_. In this case, the continuity relations at the joint between sections 2 and 3 are given as follows:

(11a) $$\phi_{2}(l_{o})=D_{0}$$

(11b) $$\phi_{3}(l_{o})=D_{0}$$

(11c) $${\frac{d\phi_{2}(x)}{dx}|}_{x=l_{o}}={\frac{d\phi_{3}(x)}{dx}|}_{x=l_{o}}$$

(11d) $${EI_{b}\frac{d^{2}\phi_{2}(x)}{dx^{2}}|}_{x=l_{o}}=EI_{b}{\frac{d^{2}\phi_{3}(x)}{dx^{2}}|}_{x=l_{o}}$$

which yield

(12a) $$A_{2}\cos k_{2}\left(l_{o}-l_{p}\right)+B_{2}\sin k_{2}\left(l_{o}-l_{p}\right)+C_{2}\cosh k_{2}\left(l_{o}-l_{p}\right)+D_{2}\sinh k_{2}\left(l_{o}-l_{p}\right)=D$$

(12b) $$A_{3}\cos0+B_{3}\sin0+C_{3}\cosh0+D_{3}\sinh0=D$$

(12c) $$\begin{array}{l} -A_{2}k_{2}\sin k_{2}\left(l_{o}-l_{p}\right)+B_{2}k_{2}\cos k_{2}\left(l_{o}-l_{p}\right)+C_{2}k_{2}\sinh k_{2}\left(l_{o}-l_{p}\right)+D_{2}k_{2}\cosh k_{2}\left(l_{o}-l_{p}\right)\\ =-A_{3}k_{2}\sin0+B_{3}k_{2}\cos0+C_{3}k_{2}\sinh0+D_{2}k_{2}\cosh0 \end{array}$$

(12d) $$\begin{array}{l} -A_{2}{k_{2}}^{2}\cos k_{2}\left(l_{o}-l_{p}\right)-B_{2}{k_{2}}^{2}\sin k_{2}\left(l_{o}-l_{p}\right)+C_{2}{k_{2}}^{2}\cosh k_{2}\left(l_{o}-l_{p}\right)+D_{2}{k_{2}}^{2}\sinh k_{2}\left(l_{o}-l_{p}\right)\\ =-A_{3}{k_{2}}^{2}\cos0-B_{3}{k_{2}}^{2}\sin0+C_{3}{k_{2}}^{2}\cosh0+D_{3}{k_{2}}^{2}\sinh0 \end{array}$$

Here, the beam has a constant displacement *D*_0_ at *x* = *l*_o_. In addition, the boundary conditions of continuous rotation angles and bending moments can be obtained at the joints between sections. By grouping Equations (3), (5), (7), and (12) together, we obtain the following eigenvalue problem for the stopper-forced bending vibration mode:

(13) $$K_{2}\left(k_{1},k_{2}\right)d={\left[\begin{array}{cccccccccccc} 0 & 0 & 0 & 0 & 0 & 0 & D_{0} & D_{0} & 0 & 0 & 0 & 0 \end{array}\right]}^{T}$$

where **K_2_** =

(14) $$\begin{array}{l} \left[\begin{array}{cccccccccccc} 1 & 0 & 1 & 0 & 0 & 0 & 0 & 0 & 0 & 0 & 0 & 0\\ 0 & k_{1} & 0 & k_{1} & 0 & 0 & 0 & 0 & 0 & 0 & 0 & 0\\ \cos k_{1}l_{p} & \sin k_{1}l_{p} & \cosh k_{1}l_{p} & \sinh k_{1}l_{p} & -1 & 0 & -1 & 0 & 0 & 0 & 0 & 0\\ -k_{1}\sin k_{1}l_{p} & k_{1}\cos k_{1}l_{p} & k_{1}\sinh k_{1}l_{p} & k_{1}\cosh k_{1}l_{p} & 0 & -k_{2} & 0 & -k_{2} & 0 & 0 & 0 & 0\\ -EI_{eq}{k_{1}}^{2}\cos k_{1}l_{p} & -EI_{eq}{k_{1}}^{2}\sin k_{1}l_{p} & EI_{eq}{k_{1}}^{2}\cosh k_{1}l_{p} & EI_{eq}{k_{1}}^{2}\sinh k_{1}l_{p} & EI_{b}{k_{2}}^{2} & 0 & -EI_{b}{k_{2}}^{2} & 0 & 0 & 0 & 0 & 0\\ EI_{eq}{k_{1}}^{3}\sin k_{1}l_{p} & -EI_{eq}{k_{1}}^{3}\cos k_{1}l_{p} & EI_{eq}{k_{1}}^{3}\sinh k_{1}l_{p} & EI_{eq}{k_{1}}^{3}\cosh k_{1}l_{p} & 0 & EI_{b}{k_{2}}^{3} & 0 & -EI_{b}{k_{2}}^{3} & 0 & 0 & 0 & 0\\ 0 & 0 & 0 & 0 & \cos k_{2}\left(l_{o}-l_{p}\right) & \sin k_{2}\left(l_{o}-l_{p}\right) & \cosh k_{2}\left(l_{o}-l_{p}\right) & \sinh k_{2}\left(l_{o}-l_{p}\right) & 0 & 0 & 0 & 0\\ 0 & 0 & 0 & 0 & 0 & 0 & 0 & 0 & 1 & 0 & 1 & 0\\ 0 & 0 & 0 & 0 & -k_{2}\sin k_{2}\left(l_{o}-l_{p}\right) & k_{2}\cos k_{2}\left(l_{o}-l_{p}\right) & k_{2}\sinh k_{2}\left(l_{o}-l_{p}\right) & k_{2}\cosh k_{2}\left(l_{o}-l_{p}\right) & 0 & -k_{2} & 0 & -k_{2}\\ 0 & 0 & 0 & 0 & -{k_{2}}^{2}\cos k_{2}\left(l_{o}-l_{p}\right) & -{k_{2}}^{2}\sin k_{2}\left(l_{o}-l_{p}\right) & {k_{2}}^{2}\cosh k_{2}\left(l_{o}-l_{p}\right) & {k_{2}}^{2}\sinh k_{2}\left(l_{o}-l_{p}\right) & {k_{2}}^{2} & 0 & -{k_{2}}^{2} & 0\\ 0 & 0 & 0 & 0 & 0 & 0 & 0 & 0 & -{k_{2}}^{2}\cos k_{2}\left(l_{b}-l_{o}\right) & -{k_{2}}^{2}\sin k_{2}\left(l_{b}-l_{o}\right) & {k_{2}}^{2}\cosh k_{2}\left(l_{b}-l_{o}\right) & {k_{2}}^{2}\sinh k_{2}\left(l_{b}-l_{o}\right)\\ 0 & 0 & 0 & 0 & 0 & 0 & 0 & 0 & EI_{b}{k_{2}}^{3}\sin k_{2}\left(l_{b}-l_{o}\right)+\omega^{2}M_{p}\cos k_{2}\left(l_{b}-l_{o}\right) & -EI_{b}{k_{2}}^{3}\cos k_{2}\left(l_{b}-l_{o}\right)+\omega^{2}M_{p}\sin k_{2}\left(l_{b}-l_{o}\right) & EI_{b}{k_{2}}^{3}\sinh k_{2}\left(l_{b}-l_{o}\right)+\omega^{2}M_{p}\cosh k_{2}\left(l_{b}-l_{o}\right) & EI_{b}{k_{2}}^{3}\cosh k_{2}\left(l_{b}-l_{o}\right)+\omega^{2}M_{p}\sinh k_{2}\left(l_{b}-l_{o}\right) \end{array}\right] \end{array}$$

We can obtain the characteristic equation by letting the determinant of **K_2_** be 0, and then solve the corresponding eigenvectors of the eigenvalues $k_{1}$ and $k_{2}$ for the vibration mode. The characteristic equation of this integrated system is a transcendental equation that yields infinitely many solutions which correspond to different eigenvalues. The solutions can be solved numerically. Furthermore, the actual mode shape can be obtained by solving the eigenvector **d**.

### 3.2. Vibration Mode Analysis

**Figure 5:**
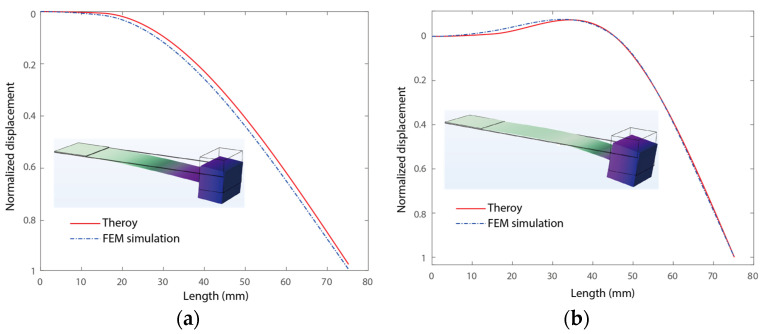
Vibration modes of the piezoelectric beam. (**a**) Without and (**b**) with hitting the stoppers.

In this section, we analyze the vibration modes of the main beam in cases with and without contact with the stoppers. We demonstrate in detail how vibration modes can up-convert the frequency of the voltage outputs, followed by finite element analyses for validation. We carried out an analysis of the vibration mode of the beam with and without hitting the stoppers, as shown in Figure 5a,b. Here, the piezoelectric transducer has dimensions of 15 × 20 × 0.2 mm^3^. The spring steel cantilever has dimensions of 75.18 × 20 × 0.16 mm^3^ with a density of 7850 kg/m^3^ and a Young’s Modulus of 200 GPa. The proof mass weighs 0.0178 kg. In addition, the two stoppers have dimensions of 45.43 × 20 × 0.6 mm^3^. The stopper’s gap D_0_ is 4 mm. The vibration modes were obtained from the modal analysis outlined in Section 3.1 and FEM simulations using COMSOL 6.0. In the FEM modal, a fixed boundary condition was applied at the fixed end of the slender piezoelectric cantilever. The other boundaries were left free for the case where the stoppers were not hit. In addition, an addition boundary condition of constant displacement at *x* = *l*_o_. Free tetrahedral meshing was adopted and modal analysis was performed.

Figure 5 shows the vibration modes of the piezoelectric beam with and without contact with the stoppers. The slender piezoelectric beam exhibited two different vibration modes in the two cases, as shown in Figure 5a,b, respectively. Here, visually large deformations were adopted to highlight the differences between the two vibration modes. In the case without contact with the stoppers, the bending yielded a positive strain on the top surface at the fixed end of the beam, as shown in Figure 5a. In this case, the piezoelectric transducer at the fixed end bore a positive strain in the longitude direction of the beam. In addition, the complicated curved vibration mode of the beam in the case of hitting the stoppers is shown in Figure 5b. Notably, the distributed displacement at the fixed end is in the opposite direction of that of the proof mass in this case. The opposite displacement yielded an opposite strain distribution on the PZT compared to that in the case without contact with the stopper. Note that the voltage output of a piezoelectric transducer is proportional to the strain distribution in the longitude direction of the beam. Therefore, the voltage output was reversed when the piezoelectric transducer hit the stopper although the direction of displacement of the beam was kept the same. In other words, hitting the stoppers changed the voltage output sign of the piezoelectric transducer in the same cycle of resonant motion. During the same cycle of motion, the slender piezoelectric cantilever maintained a low-frequency resonant motion for effective vibration absorption. In addition, the mode switching triggered the frequency-up voltage output at the same time. Three forward and back switches of the vibration mode occurred within one period of resonant motion, yielding triple up-conversion of the voltage output, as previously illustrated in Figure 2. Here, both the theoretical and numerical results are presented. The analytical results agree well with the FEM-simulated results.

## 4. Experimental Studies and Discussions

### 4.1. Experimental Set-Up

**Figure 6:**
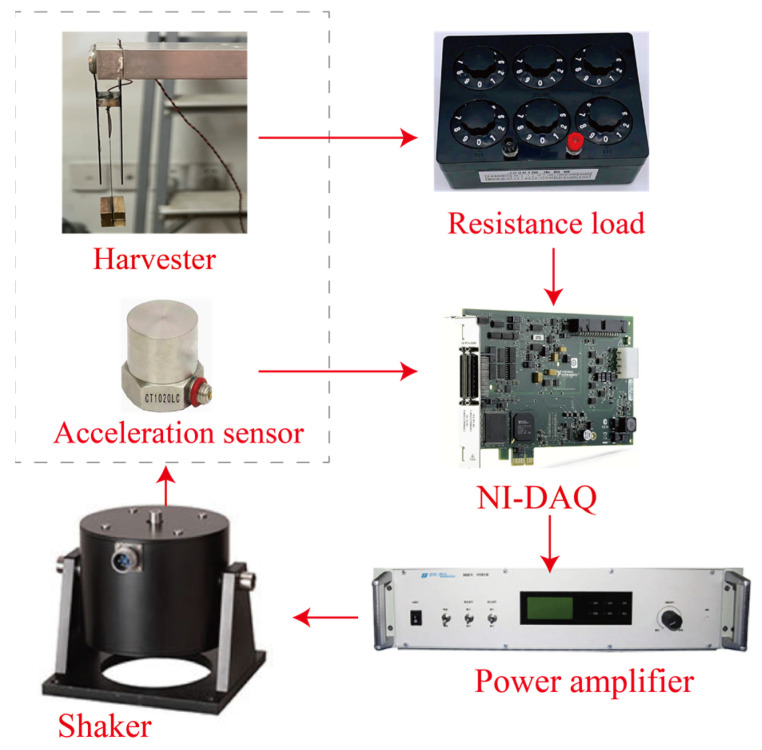
Experimental set-up.

To validate the proposed triple-frequency up-conversion mechanism, experimental studies were carried out in this section. The experimental set-up is shown in Figure 6. A piece of a piezoelectric transducer, 15 × 20 × 0.2 mm^3^, was glued at the root of the spring steel cantilever with dimensions of 75.18 × 20 × 0.16 mm^3^ using DP460 (epoxy glue) and cured for 24 h under room temperature. The lead zirconate titanate-5 piezoelectric transducers were purchased from Jiayeda, Co., Ltd., Changde, China. The piezoelectric transducer had a Young’s Modulus of 106 GPa and charge coefficient of d_31_ = −171 pC/N. The proof mass was made of four 10 × 10 × 20 mm^3^ copper blocks weighing 0.0178 kg in total. The proof mass was glued at the free end of the piezoelectric cantilever. In addition, two 45.43 × 20 × 0.6 mm^3^ spring steel beams were placed at the upper and lower sides of the piezoelectric cantilever. The gap between the main beam and the stoppers D_0_ was 4 mm. The system was mounted onto a 200 N shaker (DH40200) for the base movement excitations, purchased from Donghua, Co., Ltd., Jingjiang, China. The shaker was driven by a 2000 W power amplifier. The experiment was conducted using probes with input impedances of 100 MΩ. In addition, the signals were measured and recorded by a 16-bit NI-DAQ device (PCIe6343), purchased from National Instrument, Austin, TX, USA, with a sampling frequency of 20 kHz. In addition, the frequency sweeping signals were also generated by the same NI-DAQ device with the same sampling frequency. The root-mean-square (RMS) values of the output voltages of the piezoelectric transducers were evaluated. The amplitude of the base movement was measured by an accelerometer (CT1020LC, CHENGTEC) with a sensitivity of 200 mV/g and range of 0~25 g. In the following analysis, g = 9.8 m/s^2^ was chosen.

### 4.2. Triple Frequency Up-Conversion

**Figure 7:**
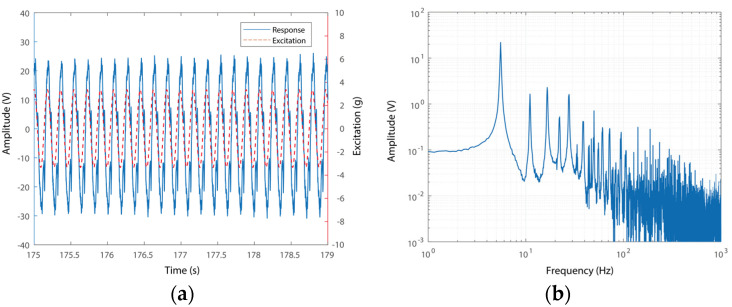
(**a**) Response and excitation and (**b**) RMS spectrum of the voltage output without triple-frequency up-conversion.

**Figure 8:**
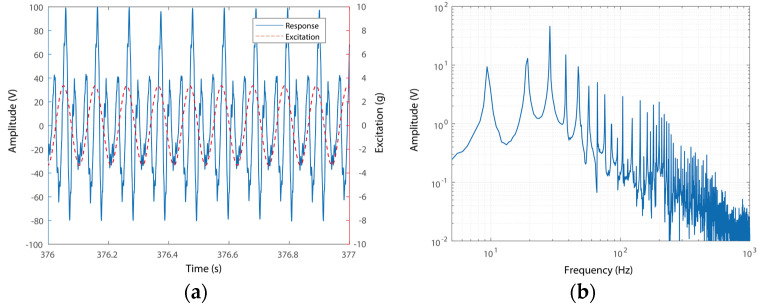
(**a**) Response and excitation and (**b**) RMS spectrum of the voltage output with triple-frequency up-conversion.

In this section, we proceed to analyze the triple-frequency up-converting voltage output of this system. Based upon the results from the experimental studies, the frequency spectrums under different scenarios were plotted and compared. Note that mode switching occurred in cases of contact between the main piezoelectric cantilever and the stoppers. Two cases were selected to illustrate this phenomenon under 3.352 g sinusoidal excitation. It is worth mentioning that the excitation was at the same level as that of human motion [49]. The first case of the voltage responses of the system was selected at around 5.54 Hz, where the main piezoelectric cantilever had slight contact with the stoppers. In addition, the second case had a voltage output of around 9.53 Hz. In the case around 9.53 Hz, the main piezoelectric cantilever hit the stoppers heavily. In this scenario, the triple-frequency up-conversion of the voltage output can be clearly observed. The time-domain responses and spectra of the two cases are presented in Figure 7 and Figure 8, respectively.

One representative time-domain signal of the responses of the voltage outputs of the PEH and the excitations is shown in Figure 7a. The responses were measured under an excitation of 5.52 Hz. The case shown in this figure indicates a conventional operation case without triple-frequency up-conversion. It can be seen from Figure 7a that the voltage output of the system has the same frequency as that of the excitation. The spectrum of the response in Figure 7b further confirms this phenomenon, i.e., the signal has a main component around 5.52 Hz, which is the same as that of the excitation. In addition, double- and triple-frequency components can be observed around 11.04 Hz and 16.56 Hz, respectively. This is because the main slender piezoelectric beam hits the stoppers in this scenario and yields high-order frequency components. In this case, the super-harmonic components have amplitudes much smaller than that of the main one. Large-amplitude triple-frequency up-conversion motion cannot be triggered due to the relatively low operational frequency and small amplitude motion. A large-amplitude triple-frequency up-conversion motion would include a new vibration mode with large stiffness, i.e., requires a higher operational frequency to trigger the resonant motion.

In addition, the time-domain signals of the voltage output and excitation of the PEH in the triple-frequency up-conversion case are shown in Figure 8a. The responses were measured under an excitation of 9.34 Hz. The 9.34 Hz value can be partly predicted by the modeling as the frequency is also determined by the nonlinear dynamics of the segmental stiffness system. This is a jumping point due to the hardening effect of the system and would move rightward under increased amplitudes of excitations. It can be seen that the voltage output of the system varies in three cycles during one excitation cycle, as shown in Figure 8a. This phenomenon indicates that the frequency of the voltage outputs has been up-converted three times. In addition, the RMS spectrum of the time-domain response can further confirm the frequency up-conversion, as shown in Figure 8b. The voltage outputs have a prominent peak at 28.59 Hz, which is three times that of the excitation. It can also be seen that the voltage output response has components of 9.34 Hz and 19.24 Hz: the basic frequency component and the double-frequency component. The two components are associated with the frequency of excitation and the motion of hitting stoppers. It is worth emphasizing that the amplitudes of the two components are much smaller than that of the tripled one. In other words, the triple-frequency responses dominate the time-domain responses. A large-amplitude triple-frequency up-conversion motion is effectively triggered due to the relatively high operational frequency and large amplitude motion. The new vibration mode has a large stiffness, i.e., yielding a higher operational frequency for the resonant motion. Thus, the efficient frequency up-conversion of the voltage output can be obtained.

**Figure 9:**
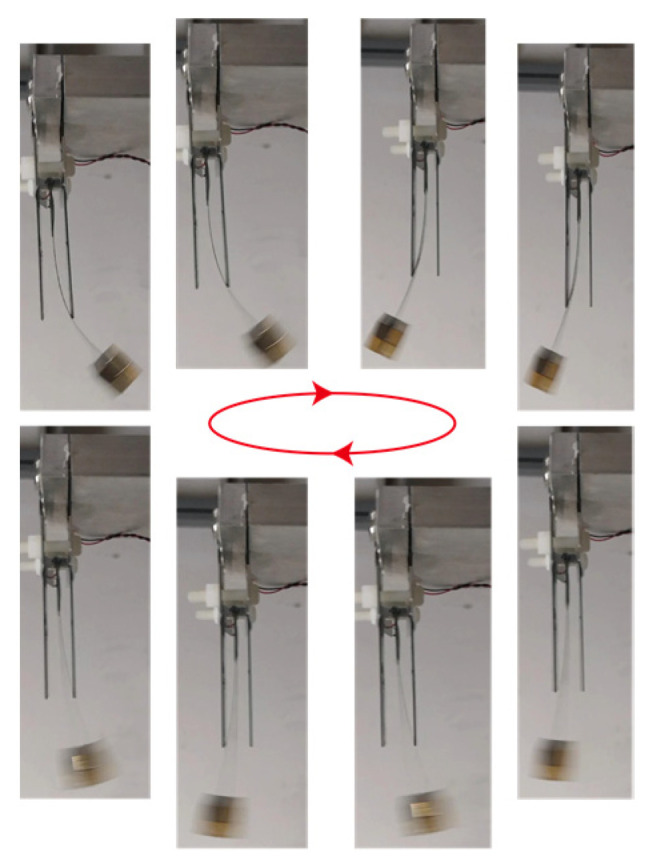
Photograph of bending mode switching (arrows mean the direction of the cycle of the motion).

Figure 9 shows the photographs of the mode switching in the experimental studies. The pictures were recorded by a cell phone with a sampling frequency of 60 Hz. It can be clearly seen that the piezoelectric beam has a pure bending mode when it does not hit the stopper. Meanwhile, its vibration mode changes into the stopper-forced complicated bending one when hitting the main beam, as detailed in Figure 2. This bending mode switching occurs multiple times during one vibration cycle, yielding a highly efficient triple-frequency up-conversion of voltage output.

### 4.3. Frequency Responses

In this section, the frequency responses of the piezoelectric beam with stoppers are plotted. Typically, voltage outputs versus excitation frequencies were plotted for evaluation of the system dynamics. Note that frequency up-conversion is highlighted in this research. In addition, the frequency components of the voltage outputs may not precisely equal to that of the excitation. Therefore, frequency response curves, as well as the time–frequency responses, were plotted for the illustration. In particular, the frequency response curves stand for the amplitude of all the frequency components of responses at a certain excitation frequency point. In addition, the time–frequency responses show in detail what the frequency components are under a certain point of the excitation frequency.

**Figure 10:**
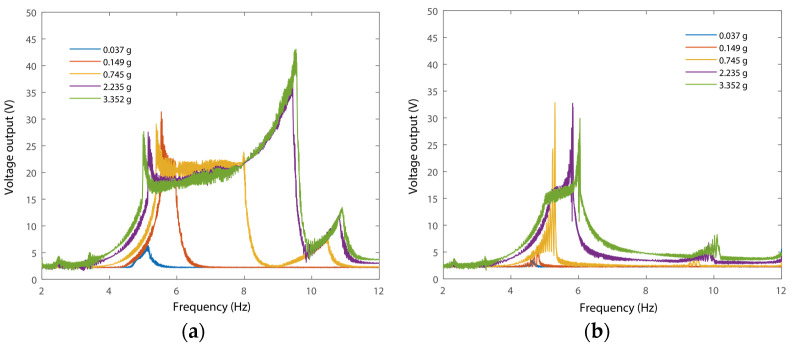
RMS voltage frequency response. (**a**) Forward and (**b**) backward frequency response.

Firstly, open-circuit voltage outputs under forward and backward frequency sweeping were measured under different amplitudes of excitations. The experimental results are presented in Figure 10.

It can be seen from Figure 10 that the frequency responses of the voltage output of the PEH show significant features of broad bandwidth in the forward frequency responses. For example, the voltage output under 3.352 g excitation has a functional range from 5 Hz to 9.52 Hz, as shown in Figure 10a. The operational bandwidth of the frequency responses is enlarged with an increase in the amplitudes of excitation. In addition, the responses under 2.235 g and 3.352 g have similar frequency responses due to the integration of stoppers. The stoppers induce a limitation of the vibration amplitudes of the system. In addition, a jumping of the frequency responses can be observed at 9.52 Hz due to the nonlinearity of the segmental stiffness of the system. The motion of hitting the stoppers yields an increased stiffness, i.e., the nonlinearity of the PEH has a hardening effect. The jumping point expanded rightward due to the hardening effect of the segmental stiffness. This is because the accumulated mechanical energy within the harvester maintained large-amplitude oscillation due to the increased equivalent stiffness of the new vibration mode. A small peak was induced around 5 Hz due to the high-order bending mode introduced. In addition, the frequency responses of the PEH decreased to a linear one as the PEH showed a small amplitude oscillation (0.037 g) without hitting the stoppers. It can also be observed that the frequency responses under forward-frequency sweeping have a significantly enlarged range compared to those under backward-frequency sweeping.

**Figure 11:**
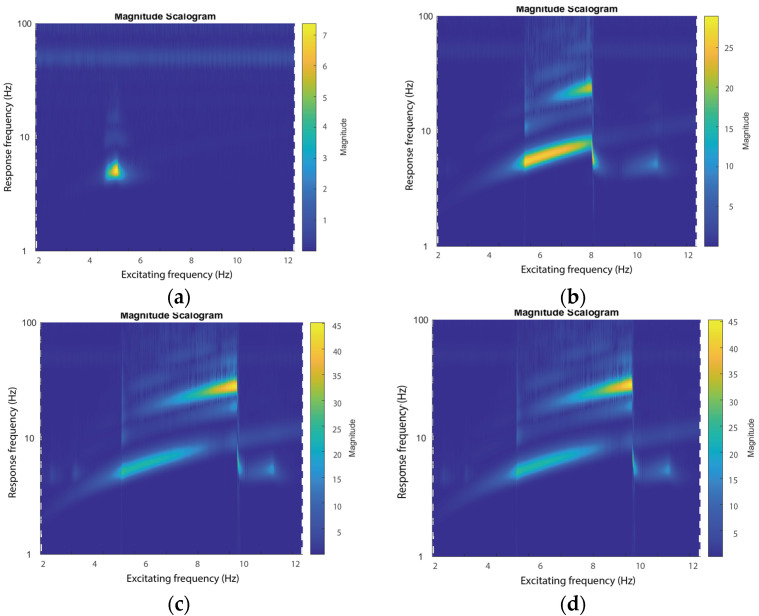
Diagrams of the frequency of excitation versus responses under excitations with amplitudes of (**a**) 0.037 g, (**b**) 0.745 g, (**c**) 2.235 g, and (**d**) 3.352 g.

In previous studies, the frequency responses of the overall voltage outputs were evaluated. On the other hand, the voltage outputs may contain many different frequency components. The frequency responses of overall voltage outputs cannot show the frequency components of the responses. Therefore, the relations between the excitation frequencies and the frequencies of the responses are calculated using wavelet transform [50], as shown in Figure 11.

The frequency up-conversion effects of voltage outputs can be obtained from the diagram showing excitation frequency versus response frequency. Figure 11 shows the diagrams of the relationship between the frequency of excitations and the frequency components of the voltage outputs. It can be observed that the frequency of the voltage outputs is essentially equal to that of the excitations when the amplitude of excitation is 0.037 g, as shown in Figure 11a. In such a case, the PEH has a small amplitude oscillation without hitting the stoppers, and the responses have little high-order frequency components. In addition, the broad bandwidth responses of the voltage outputs were obtained when the amplitude of excitations was increased to 0.745 g. The bandwidth was further expanded with an increase in the amplitudes of the excitations. For instance, large amplitude responses cover a large frequency range with excitations of 2.235 g and 3.352 g.

In addition, it can be seen that the primary frequency components dominated the responses in the vicinity of 5 Hz in all four cases. The double- and triple-frequency components had much smaller amplitudes than that of the basic one. This can be attributed to the ineffective triggering of triple-frequency up-conversion. The triple-frequency up-conversion of voltage outputs around 9 Hz becomes significant when the excitation amplitudes are increased to 2.235 g and 3.352 g, as shown in Figure 11c,d. In the two cases, the triple-frequency up-conversion of voltage outputs can be clearly obtained under excitations around 9.5 Hz, i.e., around the jumping point in the forward-frequency sweeping. This is because the hitting of the stoppers yields a hardening effect. It is also worth noticing that, in the frequency range of around 9.5 Hz, the frequency responses of voltage outputs are indeed dominated by the 28.59 Hz component due to the vibration mode switching and the voltage outputs reversing. This indicates that the main component of voltage outputs is tripled with a small basic frequency component. This phenomenon confirms the highly efficient triple-frequency up-conversion of voltage outputs, i.e., almost all the components of voltage outputs are transferred into the triple-frequency components. In addition, a small peak around 10 Hz excitation with a frequency component of 5 Hz can be observed. This peak is induced by the occasional impact induced by the stoppers. The configuration and excitation were chosen for the conceptual illustration of the new design. The frequency up-conversion can be effectively triggered under small amplitude excitation by modifying the configuration of the system, e.g., reducing the stiffness of the main beam or shrinking the distance between the two stoppers.

**Figure 12:**
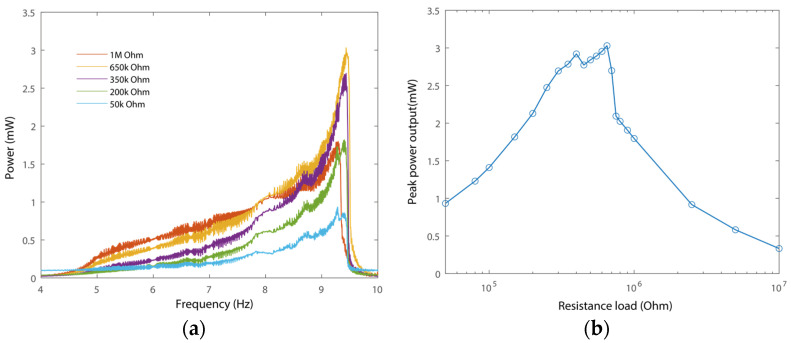
The power output performance of the inner beam with different resistance loads. (**a**) Power outputs versus frequency with different loads and (**b**) peak power outputs with different loads (circles mean data point).

Then, we evaluated the power output performance of the PEH. Here, the amplitude of the excitation was set as 3.352 g for the illustration of the power output performance, as shown in Figure 12. It can be observed that the frequency responses of power output have board bandwidth. In addition, the maximum power output, 3.03 mW, appeared when the value of the resistance load was 650 kΩ, as can be seen from Figure 12b. In addition, the maximum power output of the harvester with different resistance loads is primarily around 9.5 Hz, where triple-frequency up-conversion occurs.

It can be observed from Figure 12a that, while the voltage outputs have similar amplitudes at 9.52 Hz and 5 Hz, the power output at 9.52 Hz is significantly larger than that at 5 Hz. This is because the triple frequency up-conversion of voltage output occurs in the vicinity of 9.52 Hz. In other words, the up-converted frequency of the voltage output enables significantly enhanced power output capability around 9.52 Hz. Meanwhile, the voltage outputs have a basic frequency component of around 5 Hz. Therefore, the power output capability of the harvester remains the same as that of a conventional one, which is around 5 Hz. In such a case, the frequency response of power output has been reshaped. This phenomenon confirms the advantages of the triple frequency up-conversion.

Table 1 presents a comparison of the proposed harvester and those from previous studies. It can be obtained that the conventional ones are all based on impact trigger frequency up-conversions. In this case, the up-converted output would evaporate during every cycle of frequency up-conversion yielding limited power output capability. On the other hand, the continuous frequency up-conversion enables highly efficient power output capability. Notably, the power output capability can be effectively enhanced by integrating large-volume piezoelectric transducers.

### 4.4. Case Study of Self-Powered Sensor

**Figure 13:**
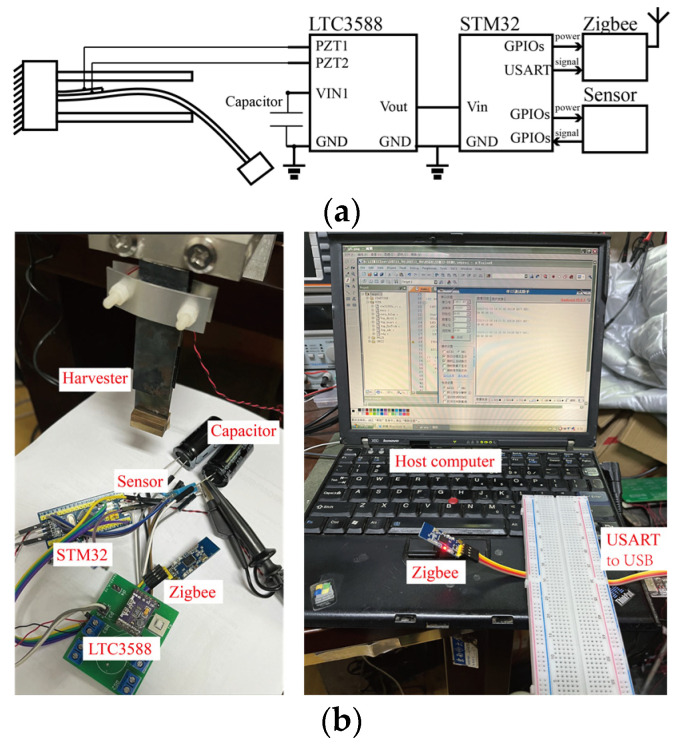
(**a**) Diagram and (**b**) experimental platform of the wireless sensing node.

In order to validate the performance of wireless self-powered sensing, a case study was then carried out. A wireless sensor node was designed, consisting of an STM32F103 microcontroller, purchased from STMicroelectronics, a humidity and temperature sensor, a Zigbee wireless transmission module, an LTC-3588 piezoelectric energy harvesting power supply, and two 4700 μF energy storage capacitors, as shown in Figure 13. To ensure sufficient and stable power supply during the operation of the wireless sensing node, in this research, large capacitors were adopted.

The STM32F103 microcontroller was utilized to control the sensor and Zigbee module. In addition, it was chosen for the demonstration of wireless self-powered sensing. Selecting a low-power microcontroller may further reduce the power consumption of the sensor node, e.g., the Attiny85 microcontroller. Notably, a wireless transmission module may consume most of the electrical energy in the circuit. It has an instantaneous power consumption of around 20 dBm (0.1 W). Therefore, we powered the wireless transmission module through multiple GPIO ports. Specifically, the GPIO ports would shut down, and the wireless transmission module would consume no power when wireless data transmission was not required.

During the majority of the time, the STM32 microcontroller was in shutdown mode to save power. A watchdog was used to wake up the microcontroller when the power was on. The sensing and wireless data transmission were triggered, and then the microcontroller was shut down again. It is worth mentioning that multiple time delay functions were integrated into the control process of the STM32-based wireless sensing node. The time delay function was adopted for waiting for the initialization and operation of the sensing and wireless transmission modules. Otherwise, errors would occur in the operation of the node.

**Figure 14:**
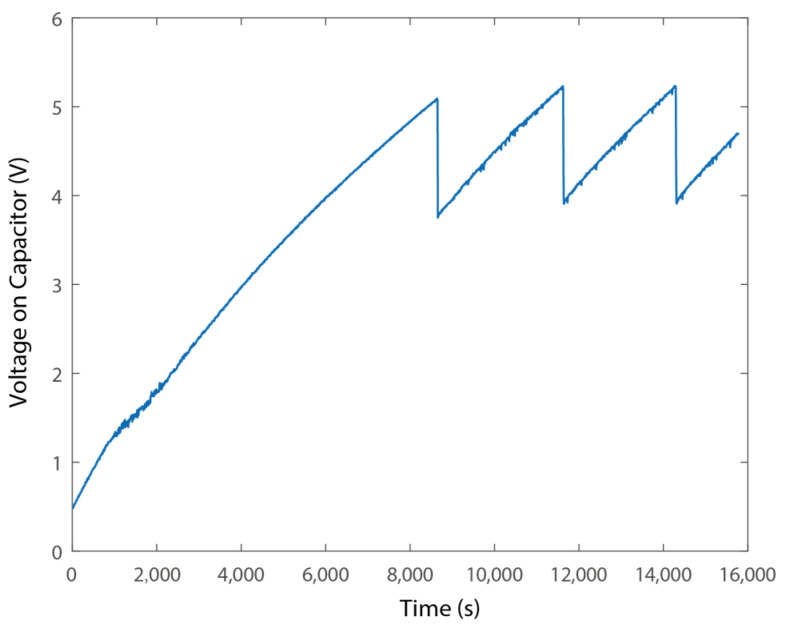
Voltage on the capacitors.

We then connected the wireless sensing node to the proposed vibration energy harvester. Figure 14 shows the periodic change in the voltage on the energy storage capacitor, representing the processing of charging and power supply to the sensor node. It can be observed that it took over 8000 s to collect sufficient electrical energy for the regular operation of the wireless sensor node. It is worth noticing also that huge capacitors were adopted. Reducing the value of the capacitor, as well as reducing the power composition of the node, can effectively reduce the time for charging. In addition, the voltage drop from 5.2 V to 3.9 V represents the power consumption of waking up, sensor data reading, and wireless data transmission. It can be determined that the operation of the microcontroller-based wireless sensing node consumes about 0.0556 J for a one-time operation.

**Figure 15:**
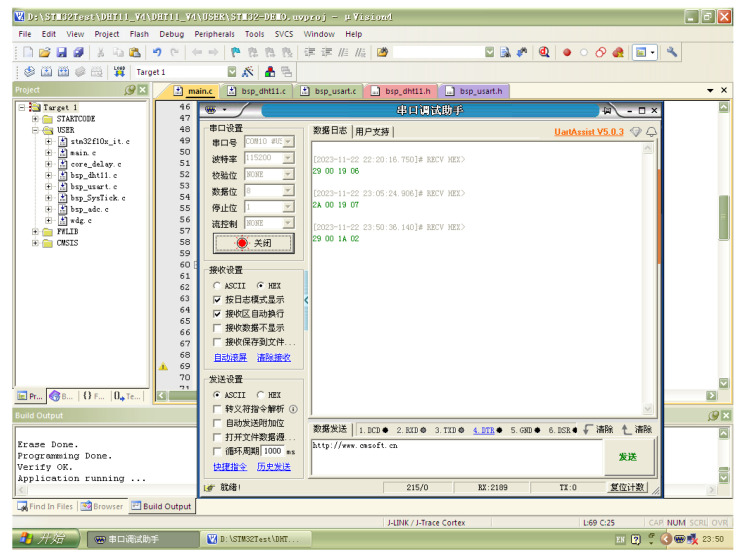
Data received by the host computer.

The received data on the host computer are shown in Figure 15. The host computer and corresponding software were always powered on for the time-uncertain data transmission of the wireless sensor node. The host computer was designed to validate the self-powered sensing capability of the proposed harvester and sensor node. It can be determined that the data can be obtained and transmitted successfully by the wireless sensing node when powered by the proposed harvester. It is worth mentioning that the designed wireless sensing node is a fully functional unit with the capabilities of edge computing, control, and wireless communication. The node consumes much more energy than a temperature–humidity meter or LED. A more advanced power management algorithm can be further designed for the optimal operation of the system. In addition, optimizations can be carried out to enhance the power output of the PEH, including of the shaping, size, and numbers of piezoelectric transducers, etc.

## 5. Conclusions

In this research, vibration-mode-switching-induced triple-frequency up-conversion of voltage output was modeled and analyzed. Two stoppers were integrated into a piezoelectric energy harvester. The stoppers provided additional boundary conditions of constant displacement that yielded a new vibration mode when the main beam contacted the stoppers. Switching between the conventional mode and the new mode yielded multiple reversals of the voltage outputs during one cycle of resonant motion. This phenomenon ultimately yielded the triple-frequency up-conversion of the voltage output. The experimental results validated the triple-frequency up-conversion. The power output performance of the PEH was analyzed and 3.03 mW power output was obtained. A wireless sensor node was designed and integrated for the validation of self-powered sensing. The optimization of the ratio of triple- and essential-frequency components will be included in future work. In addition, it is also supposed to reduce the amplitude of excitation to trigger triple-frequency up-conversion. The proposed system has potential applications in vibration-energy-harvesting-powered wireless sensing.

## Figures and Tables

**Table 1:** Comparison of different frequency up-conversion harvesters.

Refs.	Mechanism	Volume	Power	Continuous Frequency Up-Conversion
[42]	Impact-based	14 cm^3^	600 μW /42.8 μW cm^−3^	No
[45]	Impact-based	27.38 cm^3^	2.68 mW /65.74 μW cm^−3^	No
[46]	Impact-based	18.9 cm^3^	734 μW /38.8 μW cm^−3^	No
This work	Vibration mode switching	6.01 cm^3^	3.03 mW /504.16 μW cm^−3^	Yes

## Data Availability

Data will be made available on request.
